# Ingenuity Pathway Analysis of Gene Expression Profiles in Distal Nerve Stump following Nerve Injury: Insights into Wallerian Degeneration

**DOI:** 10.3389/fncel.2016.00274

**Published:** 2016-12-06

**Authors:** Jun Yu, Xiaosong Gu, Sheng Yi

**Affiliations:** Jiangsu Key Laboratory of Neuroregeneration, Co-innovation Center of Neuroregeneration, Nantong UniversityNantong, China

**Keywords:** sciatic nerve transection, distal nerve stump, microarray, bioinformatics, Ingenuity pathway analysis

## Abstract

Nerve injury is a common and difficult clinical problem worldwide with a high disability rate. Different from the central nervous system, the peripheral nervous system is able to regenerate after injury. Wallerian degeneration in the distal nerve stump contributes to the construction of a permissible microenvironment for peripheral nerve regeneration. To gain new molecular insights into Wallerian degeneration, this study aimed to identify differentially expressed genes and elucidate significantly involved pathways and cellular functions in the distal nerve stump following nerve injury. Microarray analysis showed that a few genes were differentially expressed at 0.5 and 1 h post nerve injury and later on a relatively larger number of genes were up-regulated or down-regulated. Ingenuity pathway analysis indicated that inflammation and immune response, cytokine signaling, cellular growth and movement, as well as tissue development and function were significantly activated following sciatic nerve injury. Notably, a cellular function highly related to nerve regeneration, which is called Nervous System Development and Function, was continuously activated from 4 days until 4 weeks post injury. Our results may provide further understanding of Wallerian degeneration from a genetic perspective, thus aiding the development of potential therapies for peripheral nerve injury.

## Introduction

Nerves are fragile tissues that are susceptible to traumatic injuries, such as penetration, crushing, and stretch tractions (Campbell, 2008). Nerve injury disturbs signal transmission, causes loss or alteration of sensation, impairs the power and function of target organs, and leads to disability and even mortality of victims. Therefore, it is a common and severe clinical problem worldwide.

Different from the central nervous system that can hardly regenerate by itself, the peripheral nervous system has a certain ability to regenerate on its own (Raimondo et al., 2011; Gu et al., 2014). After peripheral nerve injury, axons and their myelin sheaths in the distal nerve stump are disrupted, and Wallerian degeneration takes place. Macrophages, monocytes, and Schwann cells collectively remove axon and myelin debris and contribute to the construction of a favorable microenvironment for nerve regeneration (Brown et al., 1991, 1992; Vargas and Barres, 2007; Chen et al., 2015). Subsequently, Schwann cells in the proximal nerve stump proliferate to form the band of Bungner within the basal lamina tube, promoting the regrowth and remyelination of damaged axons, and finally leading to the regeneration of injured nerve and the reinnervation of target organs (Venezie et al., 1995; Frostick et al., 1998; Chen et al., 2007).

The Wallerian degeneration process, since its first observation by Augustus Volney Waller in 1850, has been widely studied. Over the last 160 years, however, most studies on Wallerian degeneration have been limited to morphological descriptions while molecular changes during Wallerian degeneration have not been fully elucidated (Lee and Wolfe, 2000; Zochodne, 2000; Geuna et al., 2009; Sta et al., 2014). With the development of high-throughput genomic tools, such as microarray analysis and deep sequencing, it is now possible and preferable to detect the gene expression changes during Wallerian degeneration in order to identify the molecular basis of the morphological changes.

Microarray technique provides an easy way to screen many thousands of genes or proteins in one assay, and is widely used to detect expression change patterns under various physiological and pathological conditions. In a few previous studies in our group, microarray was used to investigate the expression profiles in the distal nerve stump following peripheral nerve injury, and a number of up-regulated or down-regulated molecules were identified during Wallerian degeneration (Yao et al., 2012, 2013; Li M. et al., 2013; Li et al., 2014). Furthermore, many statistical and bioinformatic tools, including Hierarchical clustering, Euclidean distance matrix, Venny plot analysis, Volcano plot analysis, principal component analysis, Gene Ontology analysis, and Kyoto Enrichment of Genes and Genomes pathway analysis, have been applied to determine key molecules, signaling pathways, and biological processes during Wallerian degeneration. For example, Gene Ontology analysis suggested that differentially expressed genes in the distal nerve stump could be mainly divided into functional groups with regulatory functions, including cell communication, cell transport, and transcriptional regulation (Bosse et al., 2006). Biological processes, such as response to stimulus, inflammatory response, immune response, cell proliferation, migration, and apoptosis, axon guidance, myelination, signal transduction, and protein kinase activity, were also investigated (Jiang et al., 2014). Despite these findings, it is still required to further investigate the molecular changes of Wallerian degeneration from a genetic perspective.

Fortunately, the progress in bioinformatic analysis allows us to better interpret the gene expression profiles as revealed by microarray. One of advanced bioinformatic tools is the Ingenuity pathway analysis (IPA) software program, which can analyze the gene expression patterns using a build-in scientific literature based database (according to IPA Ingenuity Web Site, www.ingenuity.com). In the current study, we made rat sciatic nerve transection, collected the distal nerve stump samples at different time points, re-analyzed previous obtained microarray data (Yao et al., 2012, 2013) by using the R software and the limma package and uploaded the massive microarray data into the IPA program for a systematic bioinformatic analysis. The application of IPA analysis helps to decipher dynamic molecular changes in the distal part of injured sciatic nerve, especially in Schwann cells and macrophages, two important types of cells in the distal stump, and thus may provide some new knowledge about Wallerian degeneration.

## Experimental procedures

### Animal surgery and sample preparation

All animal procedures were ethically approved by the Administration Committee of Experimental Animals, Jiangsu Province, China and were used in accordance with Institutional Animal Care guideline of Nantong University. Adult male Sprague-Dawley (SD) rats, body weight 180–200 g, were purchased from the Experimental Animal Center of Nantong University and housed in temperature- and humidity-controlled large cages with sawdust bedding and given access to tap water and food *ad libitum*. Rats were randomly divided into 11 groups according to different observation points and went through surgical transection of sciatic nerves as previously described (Yu et al., 2012). Briefly, rats were anesthetized by an injection of mixed narcotics (85 mg/kg trichloroacetaldehyde monohydrate, 42 mg/kg magnesium sulfate, and 17 mg/kg sodium pentobarbital). Rat sciatic nerve was exposed through an incision on the lateral aspect of the mid-thigh of the left hind limb and a 10-mm nerve segment was excised. At 0.5, 1, 6, 12, and 24 h, 4 days, and 1, 2, 3, and 4 weeks after surgery, rats were sacrificed by decapitation. The distal nerve stumps were removed and stored at −80°C. The rats in the 0 h group received sham-surgery on their left sciatic nerves were used as controls.

### Microarray data analysis

Stored distal nerve stumps were used for total RNA isolation. Total RNA was extracted with Trizol reagent (Life technologies, Carlsbed, CA) and cleaned with RNeasy spin columns (Qiagen, Valencia, CA). The quality and quantity of isolated RNA were checked by Agilent Bioanalyzer 2100 (Agilent technologies, Santa Clara, CA) and NanoDrop ND-1000 spectrophotometer (Infinigen Biotechnology Inc., City of Industry, CA), respectively. Microarray analysis was performed by an Affymetrix GeneChip Hybridization Oven 640 and Gene Array Scanner 3000. Microarray data analysis was performed by the R software platform (v.2.13.0) and the limma (linear regression model) package (Ritchie et al., 2015; Xu and Sun, 2015). Detected global gene expressions at each time point following sciatic nerves transection were compared with the control group. Genes with a fold change greater than 2 (the absolute value of log_2_ fold change greater than 1) and an adjust *p*-value less than 0.05 were considered as differentially expressed and were investigated by IPA (Ingenuity Systems Inc., Redwood City, CA).

### Bioinformatic analysis

Bioinformatic analysis was performed to analysis differentially expressed genes at 0.5, 1, 6, 12, and 24 h, 4 days, and 1, 2, 3, and 4 weeks post sciatic nerve transection. Briefly, outcomes from microarray analysis were first uploaded into Qiagen's IPA system for core analysis and then overlaid with the global molecular network in the Ingenuity pathway knowledge base (IPKB). IPA was performed to identify canonical pathways, diseases and functions, and gene networks that are most significant to microarray outcomes and to categorize differentially expressed genes in specific diseases and functions. Heatmap and hierarchical cluster analysis were used to demonstrate the expression patterns of these differentially expressed genes.

### Quantitative real time polymerase chain reaction (qPCR)

RNA samples were converted to cDNA using the Prime-Script reagent Kit (TaKaRa, Dalian, China). Obtained cDNA was used as a template for amplification and qPCR was performed using SYBR Green Premix Ex Taq (TaKaRa) with specific primer pairs on an Applied Biosystems Stepone real-time PCR System. The sequences of primer pairs were listed in Supplementary Table 1. The thermal setting was 5 min at 95°C; 40 cycles of 30 seconds at 95°C, 45 seconds at the annealing temperature, 30 seconds at 72°C; and 5 min at 72°C. Relative quantification of mRNA was conducted using the comparative 2^−ΔΔCt^ method with GAPDH as the reference gene, in which ΔCt = Ct_(post injury)_ − Ct_(control)_, ΔΔCt = ΔCt_(Target gene)_− ΔCt_(GAPDH)_. The quality of qPCR was examined by a single peak melt curve corresponding to single PCR product. qPCR outcomes were expressed as means ± SEM, and analyzed by means of SPSS 15.0 software (SPSS, Chicago, IL). A *p*-value less than 0.05 was considered as significantly different.

## Results

### Overview of genomic responses in distal sciatic nerve stump

**Figure 1:**
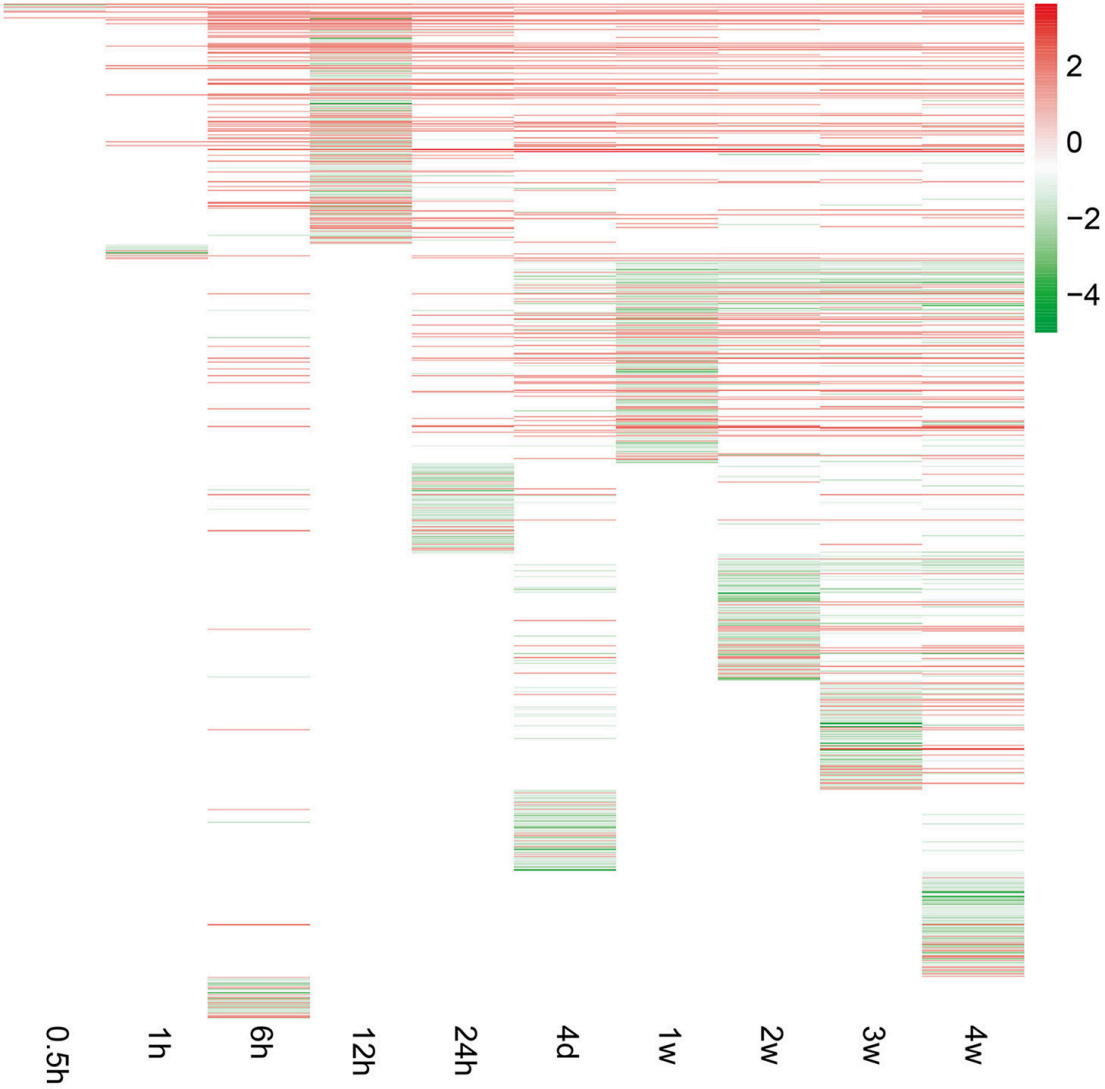
**Gene expression patterns at each time point following sciatic nerve transection**. The expression levels of genes were indicated by the color bar (attached to the raster plot). Red color indicates up-regulation while green color indicates down-regulation.

To investigate peripheral nerve injury-induced molecular changes during Wallerian degeneration, microarray analysis was performed to detect the expression levels of more than 30,000 genes at 0, 0.5, 1, 6, 12, and 24 h, 4 days, and 1, 2, 3, and 4 weeks in the distal nerve stumps post sciatic nerve transection. Compared with the expressed genes at 0 h post injury, only a small number of genes were differentially expressed at 0.5 and 1 h after nerve injury. The number of differentially expressed genes increased starting from 6 h post injury and remained at a high level at 4 weeks post injury (Figure 1).

### Canonical pathway analysis

**Figure 2:**
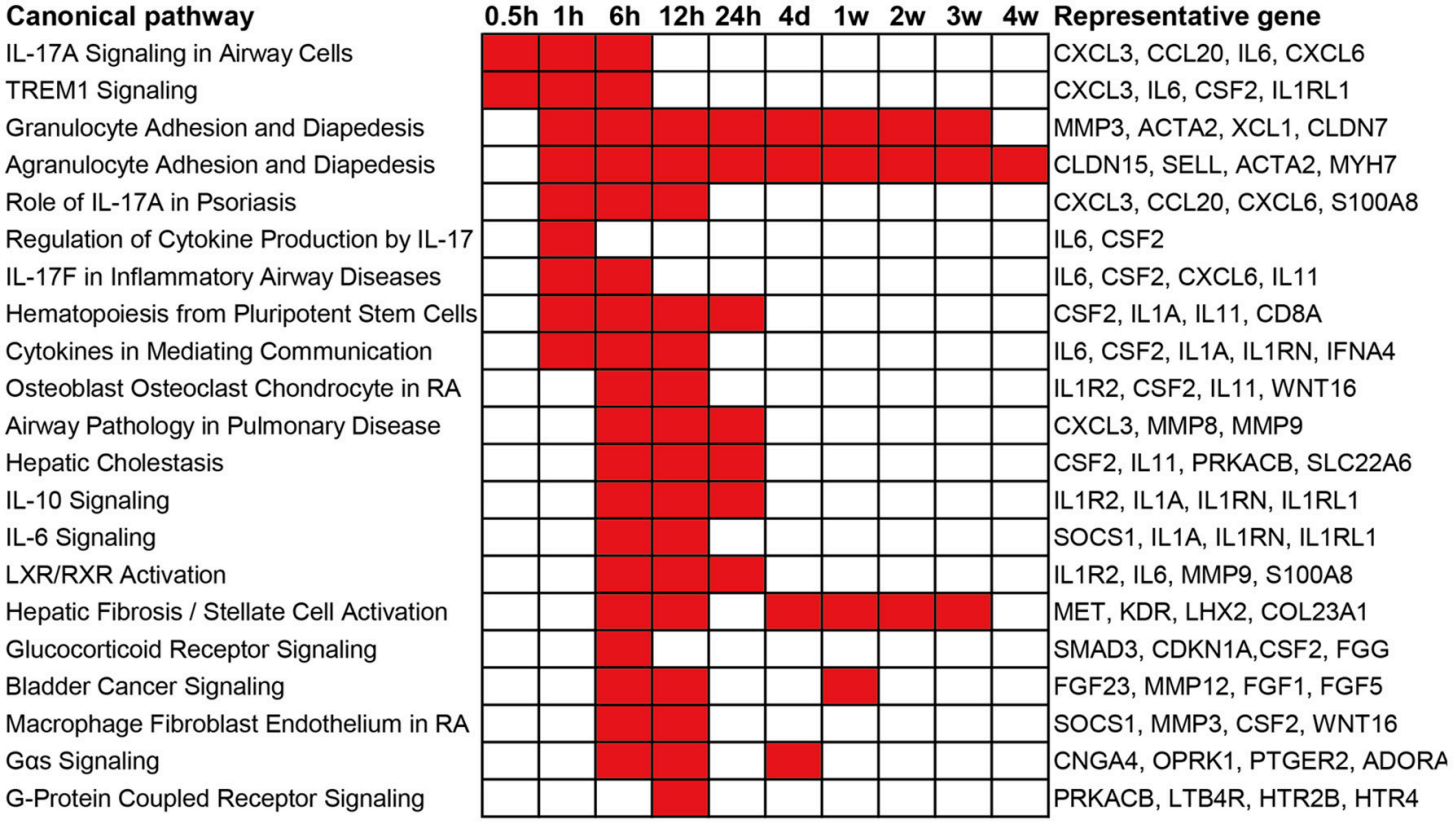
**Top enriched canonical pathways following sciatic nerve transection**. Canonical pathways with a *p*-value < 10^−3^ (1E-03) are labeled in red color while canonical pathways with a *p*-value > 10^−3^ (1E-03) are labeled in white color. The representative differentially expressed genes in the canonical pathway are listed to the right.

To obtain an elementary investigation of the molecular mechanisms underlying Wallerian degeneration, microarray data were submitted to IPA core analysis. The differentially expressed genes were categorized to related canonical pathways based on IPKB. The top enriched categories of canonical pathways with a *p*-value less than 10^−3^ as well as representative differentially expressed genes in each canonical pathway are listed in Figure 2. At 0.5 h post sciatic nerve transection, only Interleukin-17A (IL-17A) Signaling in Airway Cells (an IL-17 cytokine signaling) and TREM1 Signaling (a proinflammatory immune response) are significantly activated. At 1 h post nerve transection, canonical pathways related to immune response and cytokine signaling were activated. Starting from 6 h post injury, a larger number of canonical pathways were activated, which were mainly related to inflammation and immune response, cytokine signaling, diseases, nuclear receptor signaling, and intracellular and second messenger signaling. From 4 days until 4 weeks post injury, less canonical pathways were activated. Only a signaling pathway, called Agranulocyte Adhesion and Diapedesis, was activated during nearly whole post-injury period. All categories of canonical pathways and their associated genes are listed in Supplementary Table 2.

### Disease and function analysis

**Figure 3:**
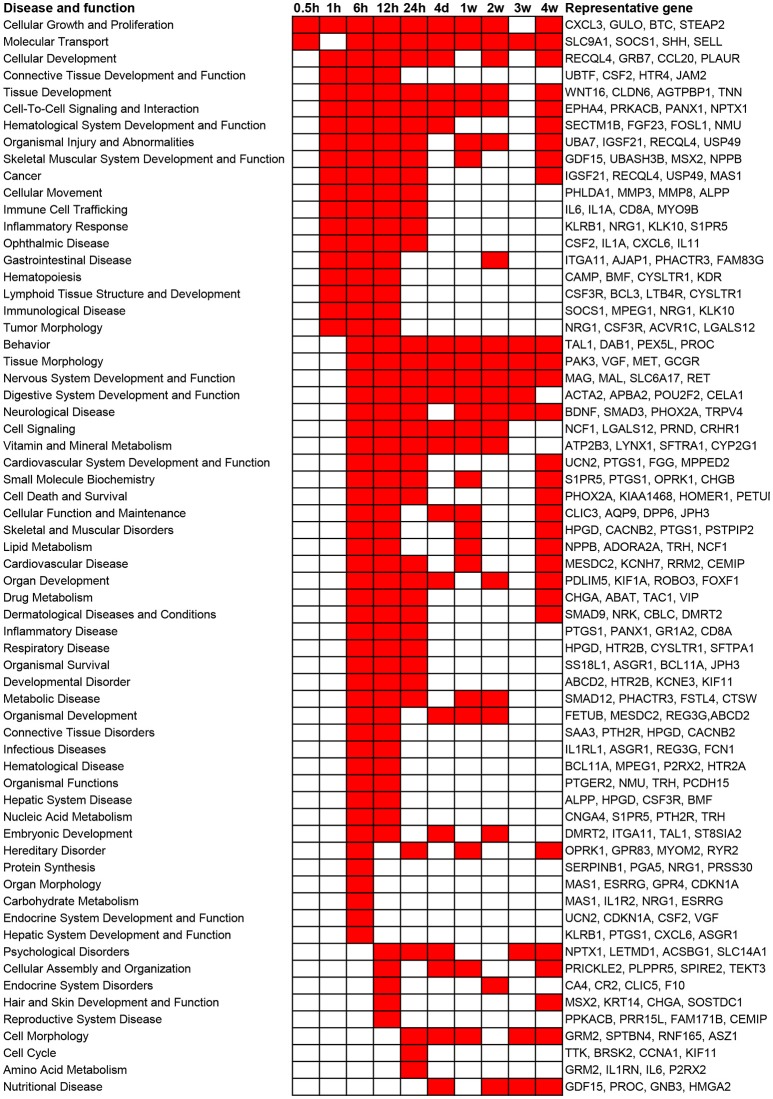
**Top enriched diseases and functions following sciatic nerve transection**. Diseases and functions with a *p*-value < 10^−5^ (1E-05) are labeled in red color while diseases and functions with a *p*-value > 10^−5^ (1E-05) are labeled in white color. The representative differentially expressed genes in the disease and function are listed to the right.

In addition to canonical pathways, differentially expressed genes were also categorized to related diseases and functions. Similar as the results from canonical pathway analysis, diseases and functions with a *p*-value less than 10^−5^ as well as involved representative genes are listed in Figure 3. Consistent with the results of canonical pathway analysis, following sciatic nerve transection, the number of categories of diseases and functions first increased and then progressively declined. Only cellular functions of Cellular Growth and Proliferation and Molecular Transport were considerably activated at 0.5 h post injury. At 1 h post injury, significant activated cellular functions were generally related to inflammation and immune response, tissue development, and numerous diseases. At 6 to 24 h post injury, diseases and cellular functions related to organ development, tissue and organ morphology, tissue disorder, as well as organismal survival and function were significantly activated. At 4 days to 4 weeks post injury, which represented a sub-chronic injury stage, less numerous categories of diseases and functions were involved. Notably, a cellular function called Nervous System Development and Function was drastically stimulated at 6, 12, and 24 h post injury, and was enriched during nearly whole post-injury period. All categories of diseases and functions and their associated genes are listed in Supplementary Table 3.

### Gene network analysis

Besides the predominant pathways and cellular functions, gene networks were built to connect key genes and enriched categories of diseases and functions based on the correlation between differentially expressed genes. Gene networks and their related top diseases and functions are listed in Supplementary Table 4.

At 0.5, 1, 6, 12, and 24 h post nerve injury, top networks had scores of 21, 23, 23, 27, and 29, respectively. These top networks were mainly connected to the functions: Cellular Movement and Cell-To-Cell Signaling. From 4 days post nerve injury, the scores of top networks were even higher and networks with a score value higher than 30 are presented in Table 1. These top networks are generally associated with cellular morphology, assembly, organization, and organ development.

**Table 1:** **Top enriched networks following sciatic nerve transection**.

**4 days**	
1	Skeletal and muscular system development and function, developmental disorder, neurological disease
2	Cell signaling, nucleic acid metabolism, small molecule biochemistry
**1 week**	
1	Organ development, nervous system development and function, cardiovascular disease
**2 weeks**	
1	Developmental disorder, hereditary disorder, ophthalmic disease
2	Behavior, cellular function and maintenance, nervous system development and function
**3 weeks**	
1	Cell morphology, cellular assembly and organization, nervous system development and function
2	Reproductive system development and function, cell cycle, cell-mediated immune response
**4 weeks**	
1	Neurological disease, psychological disorders, cell-to-cell signaling and interaction
2	Cell-to-cell signaling and interaction, nervous system development and function, neurological disease
3	Digestive system development and function, embryonic development, organ development

*Networks at each time point following sciatic nerve transection with a score >30 are listed in the table*.

It is worth noting that cellular function Nervous System Development and Function was vastly involved in many gene networks at nearly all tested time points, except at 0.5 and 1 h post injury (Table 2). Therefore, genes involved in this function category were further analyzed.

**Table 2:** **The involvement of Nervous System Development and Function at different time points**.

**Time point**	**Network rank**	**Score**
6 h	2	23
	5	19
12 h	8	17
	11	15
24 h	1	29
	2	27
	7	2
	11	2
4 days	14	2
1 week	1	40
	6	24
2 weeks	2	37
	6	26
3 weeks	1	39
	6	21
	15	2
4 weeks	8	23

*The involved cellular function of Nervous System Development and Function in gene network at different time points following sciatic nerve transection are listed in the table*.

### Nervous system development and function

**Figure 4:**
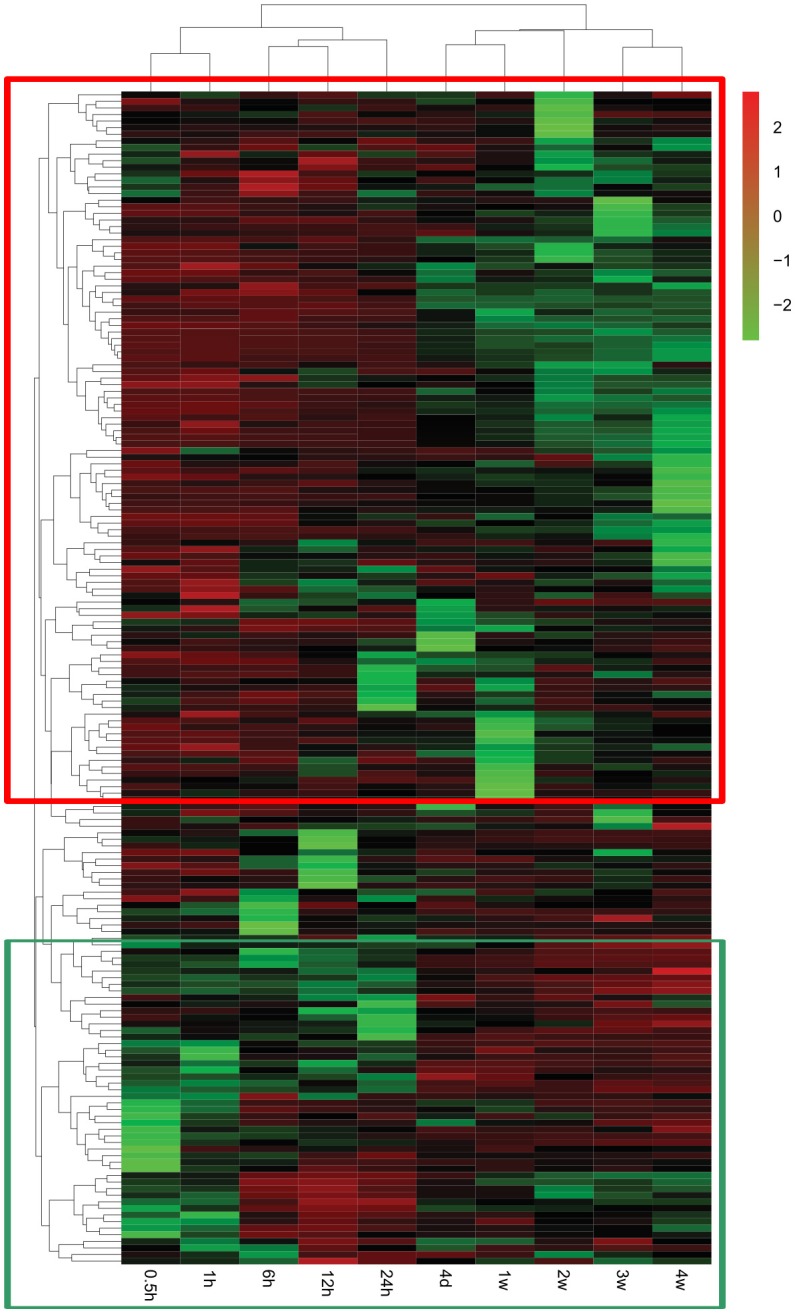
**Heatmap and hierarchical clustering of genes involved in Nervous System Development and Function**. The expression levels of genes were indicated by the color bar above the heatmap. Red color indicates the increased expression whereas green color indicates the decreased expression as compared to control (expression at 0 h). Genes that were first up-regulated and then down-regulated are boxed in the red frame while genes that were first down-regulated and then up-regulated are boxed in the green frame.

A global genome heatmap with hierarchical cluster analysis demonstrated the gene expression profiles in Nervous System Development and Function (Figure 4). The heapmap suggested that nearly 2/3 of genes involved in this cellular function were firstly up-regulated and then down-regulated while a little bit less than 1/3 of differentially genes were first down-regulated and then up-regulated. The differentially expressed genes involved in Nervous System Development and Function are listed in Supplementary Table 5.

**Figure 5:**
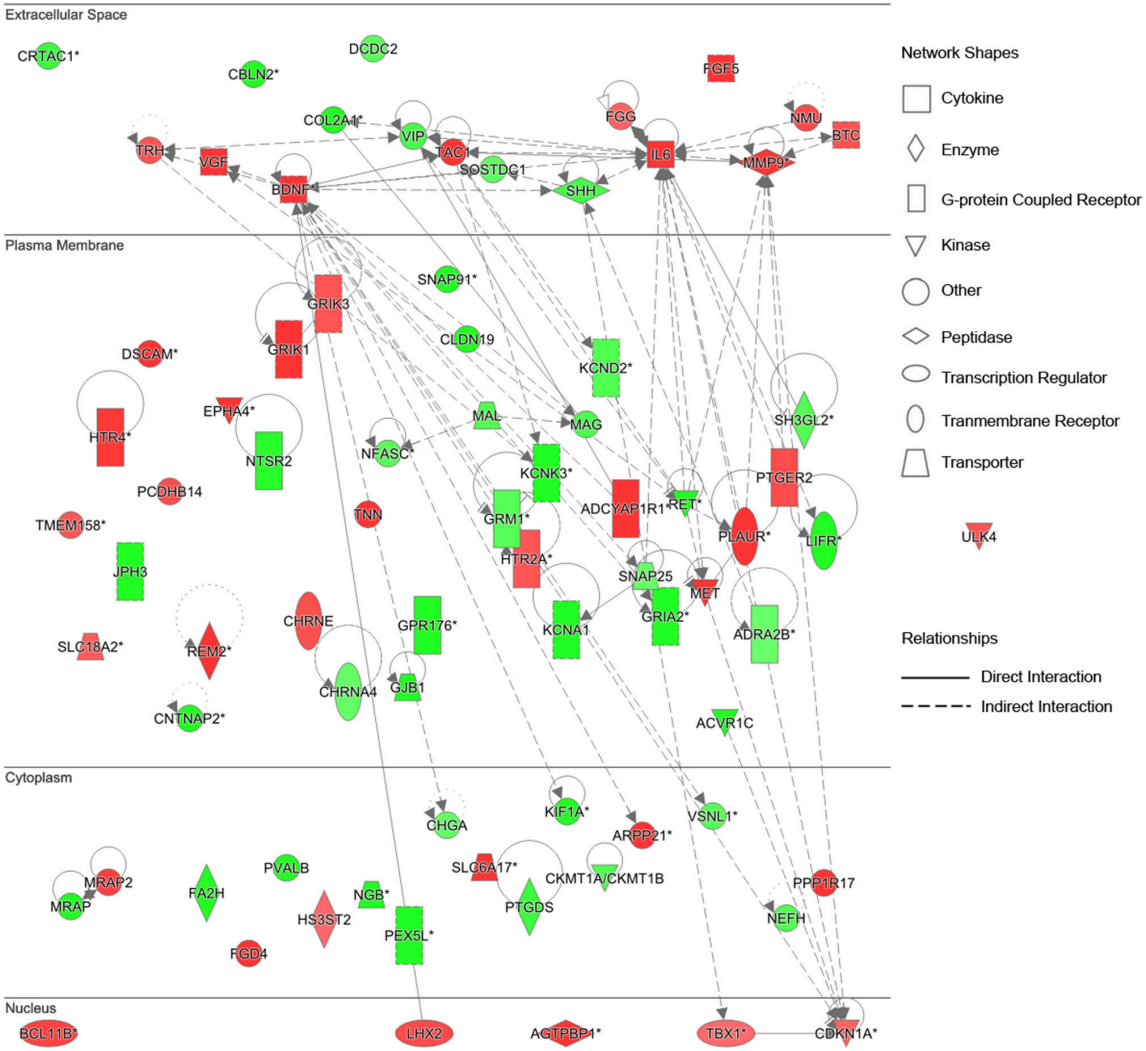
**Differentially expressed genes in Nervous System Development and Function at 4 weeks post sciatic nerve transection**. Differentially expressed genes involved in nervous system development and function at 4 weeks post sciatic nerve transection are listed in the network. The brightness of color is related to the fold change of differentially expressed gene and the darker the color, the higher fold change.

To further identify key genes involved in this cellular function and establish the connections in-between these genes, the gene network diagrams were made based on IPKB. Among these gene network diagrams, a diagram related to 4 weeks post nerve injury is shown in Figure 5, and other diagrams related to other time points are shown in Supplementary Figure 1. As indicated by all diagrams, IL-6 was immediately up-regulated at 0.5 h post nerve injury and highly expressed at all tested time points; with the passage of time, more genes were differentially expressed and the molecular network seemed more complex. For instance, matrix metalloproteinase 9 (MMP9) and brain derived neurotrophic factor (BDNF) were drastically up-regulated starting from 6 h post injury and remained at high expression levels until 4 weeks post injury. Sonic hedgehog (SHH) was first up-regulated at 6 h post injury and then down-regulated at later time points. Myelin-associated glycoprotein (MAG) and myelin and lymphocyte protein (MAL) were down-regulated at 4 days and 1 week post nerve injury, respectively. In addition, the gene network diagram suggested that these differentially expressed genes were not independent but were highly correlated with each other.

**Figure 6:**
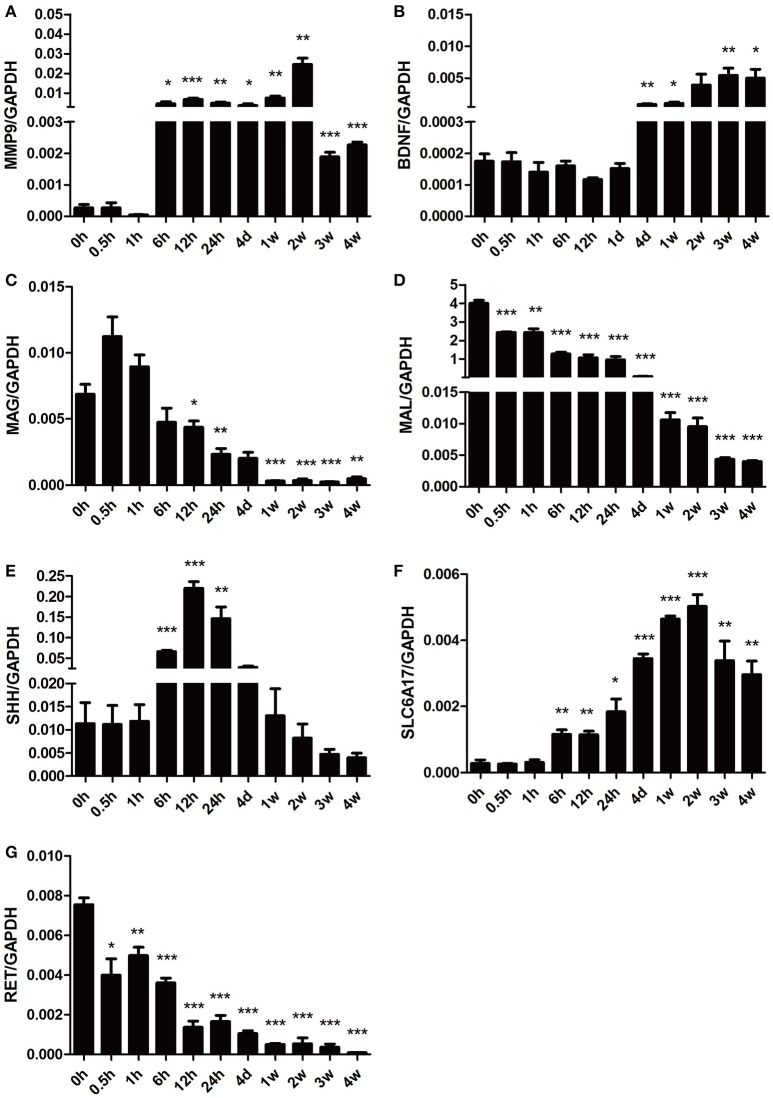
**qPCR analysis of genes involved in Nervous System Development and Function following sciatic nerve transection**. The relative expression levels of **(A)** MMP9, **(B)** BDNF, **(C)** MAG, **(D)** MAL, **(E)** SHH, **(F)** SLC6A17, and **(G)** RET were calculated using comparative Ct with GAPDH as the reference gene. Data are summarized from 3 independent experiments and values are shown as the means ± SEM. The asterisk indicates significant difference: ^*^p-value < 0.05; ^**^p-value < 0.01; ^***^p-value < 0.001 as compared to contrsol (0 h post injury).

To further validate the gene expression profiles identified by microarray analysis, some representative differentially expressed genes involved in Nervous System Development and Function were selected for qRT-PCR examination (Figure 6). Consistent with the gene network diagram, MMP9 and BDNF were up-regulated post nerve injury while MAG and MAL were down-regulated. The expression patterns of SHH, solute carrier family 6 member 17 (SLC6A17), and ret proto-oncogene RET were also in agreement with microarray results.

## Discussion

In this study we used microarray and bioinformatic analysis to examine dynamic molecular changes in the distal nerve stump following peripheral nerve injury. IPA results provided us with some new insights into Wallerian degeneration.

Immediately after peripheral nerve injury, the most significant pathways and cellular functions were found to be inflammatory and immune response, which remained to be activated until 4 weeks post nerve injury. Meanwhile, immune signal molecules and cytokines were also activated rapidly after injury. Inflammation reaction and immune response are protective reactions against injury stimuli (Donnelly and Popovich, 2008; Benowitz and Popovich, 2011; Dubový et al., 2013; Li S. et al., 2013). Timely inflammatory and immune reactions are highly relevant with Wallerian degeneration, and benefit subsequent nerve repair and functional recovery (Dubový, 2011; Gaudet et al., 2011). Morphological observation of Wallerian degeneration suggests that macrophages migrate to the injured site in an early stage post injury (Geuna et al., 2009). Our study, from the genetic aspect, confirmed the critical roles of macrophages during Wallerian degeneration. Moreover, our finding that inflammation and immune response was continuously activated is consistent with previous observation in a model of sciatic nerve crush injury (Yi et al., 2015).

At relatively later time points post peripheral nerve injury, the most significant canonical pathways and cellular functions were found to be tissue development and function. The IPA-derived gene network suggested that the category of Nervous System Development and Function was highly scored from 6 h to 4 weeks post nerve injury. The examination for the genes involved in Nervous System Development and Function indicated that MMP9 and BDNF were significantly up-regulated starting from 6 h post nerve injury. MMP9, a gelatinase, belongs to the MMP zinc-containing endopeptidase family. During tissue remodeling, MMPs mediate the breakdown of the extracellular matrix (ECM), a key network containing physical and biochemical cues for tissue regeneration (Ravanti and Kähäri, 2000; Dubový, 2004; Chen et al., 2007). Previous studies also revealed that the MMP9 expression was often elevated following nerve injury. However, the up-regulation of MMP9 was largely compromised in mice with delayed Wallerian degeneration (Shubayev et al., 2006; Barrette et al., 2010). Up-regulated MMP9 stimulates the recruitment and migration of macrophages, mediates the differentiation of myelinating Schwann cells, and thus benefits nerve regeneration (Shubayev et al., 2006; Kim et al., 2012). Our earlier studies also demonstrated that two other members of the MMP family, MMP7 and MMP12, were kept up-regulated following nerve injury (Jiang et al., 2014; Qin et al., 2016). All these results highlight the central roles of the MMP family in nerve regeneration. BDNF is a well-known neurotrophic factor that promotes neuronal survival and activity and stimulates axon growth (Braun et al., 1996; Lykissas et al., 2007). We have previously reported on the up-regulation of the mRNA expression of BDNF at 1, 4, 7, and 14 days post nerve crush injury (Yi et al., 2016). It has been known that after peripheral nerve injury, Schwann cells not only proliferate to form the bands of Bungner, but also produce a range of neurotrophic factors, including BDNF (Frostick et al., 1998; Faroni et al., 2013; Jang et al., 2016). Therefore, the up-regulated BDNF in the distal nerve stump would augment axonal regrowth and promote nerve regeneration. In this study, besides MMP9 and BDNF, myelin-related genes (MAG and MAL) were also found to be up-regulated starting from 4 days post injury. Myelin is an important inhibiting factor for neurite growth and nerve repair (Bähr and Przyrembel, 1995; David et al., 1995). Why were not the myelin-related genes up-regulated until a relatively later stage post injury? It may be because that at an early stage after nerve injury only non-myelinating Schwann cells, but not myelinating Schwann cells, enter into the cell cycle to avoid negative factors for nerve regeneration (Murinson et al., 2005), but later on myelinating Schwann cells start to proliferate and to form myelin sheaths (Liu et al., 1995; Vargas and Barres, 2007). Accordingly, the expression change of myelin-related genes might reflect that Schwann cells play different roles at different time periods during Wallerian degeneration.

Following sciatic nerve transection, a diverse array of biological processes, including stimulus detection and response, inflammatory and immune response, cell migration, cell proliferation, cell death, axonal regeneration and guidance, and myelination, were significantly activated in the proximal nerve stump (Li S. et al., 2013). In contrast, many of these biological processes were also detected in the distal nerve stump after peripheral nerve injury, as revealed by this study. Certainly, the gene expression patterns and activated biological processes in the distal nerve stump showed different characteristics compared to those in the proximal nerve stump. There has been a previous study which compared the differentially gene expressions in proximal and distal nerves after nerve injury (Jiang et al., 2014). It should be known that the proximal and the distal nerve stumps may share many common genes but have different spatiotemporal expression patterns after nerve injury. Further in-depth studies are needed to determine which genes and functions represent the key molecular elements for peripheral nerve regeneration.

## Conclusions

In this study, we applied microarray to identify differentially expressed genes in the distal nerve stumps after sciatic nerve transection, and further used IPA program as the main bioinformatic tool to analyze microarray data. An integrated global view of gene expression patterns during Wallerian degeneration was obtained by performing IPA core analysis, overlaying microarray data to IPKB, and then assigning differentially expressed genes to specific canonical pathways, diseases and biological functions, and networks. The inflammation and immune response, cytokine signaling, cellular growth and movement, and, especially, Nervous System Development and Function, were significantly activated in the distal nerve stump post peripheral nerve injury, suggesting their critical roles in Wallerian degeneration. Our results may help to illuminate the molecular aspects of Wallerian degeneration and to develop potential therapeutic targets for peripheral nerve repair.

## Author contributions

Conceived and designed the experiments: JY, XG and SY. Performed the experiments: JY and SY. Analyzed the data: JY and SY. Contributed reagents/materials/analysis tools: JY and SY. Wrote the manuscript: SY. All authors have read and approved the final manuscript.

### Conflict of interest statement

The authors declare that the research was conducted in the absence of any commercial or financial relationships that could be construed as a potential conflict of interest.

## Supplementary material

Supplementary Table 1 **List of primer pairs for real-time quantitative qPCR**.

Supplementary Table 2 **List of all categories of canonical pathways and involved molecules following sciatic nerve transection**.

Supplementary Table 3 **List of all categories of diseases and biological functions and involved molecules following sciatic nerve transection**.

Supplementary Table 4 **List of the gene networks at 0.5, 1, 6, 12, and 24 h, 4 days, and 1, 2, 3, and 4 weeks post sciatic nerve transection**.

Supplementary Table 5 **List of differentially expressed genes involved in Nervous System Development and Function following sciatic nerve transection**.

Supplementary Figure 1 **Differentially expressed genes involved in Nervous System Development and Function at 0.5, 1, 6, 12, and 24 h, 4 days, and 1, 2, and 3 weeks post sciatic nerve transection**.

## Abbreviations

IPA: Ingenuity pathway analysis
SD: Sprague-Dawley
IPKB: Ingenuity pathway knowledge base
qPCR: quantitative real time polymerase chain reaction
IL: Interleukin
MMP: matrix metalloproteinase
BDNF: brain derived neurotrophic factor
SHH: sonic hedgehog
MAG: myelin-associated glycoprotein
MAL: myelin and lymphocyte protein
SLC6A17: solute carrier family 6 member 17
ECM: extracellular matrix.
