# Supplementary material for: Ingenuity Pathway Analysis of Gene Expression Profiles in Distal Nerve Stump following Nerve Injury: Insights into Wallerian Degeneration
Source: Front Cell Neurosci. 2016 Dec 6;10:274. doi: 10.3389/fncel.2016.00274 (PMC5138191; doi:10.3389/fncel.2016.00274)
Supplement: Supplementary Figure 1 — Differentially expressed genes involved in Nervous System Development and Function at 0.5, 1, 6, 12, and 24 h, 4 days, and 1, 2, and 3 weeks post sciatic nerve transection. [file Image1.pdf]

0.5 hour

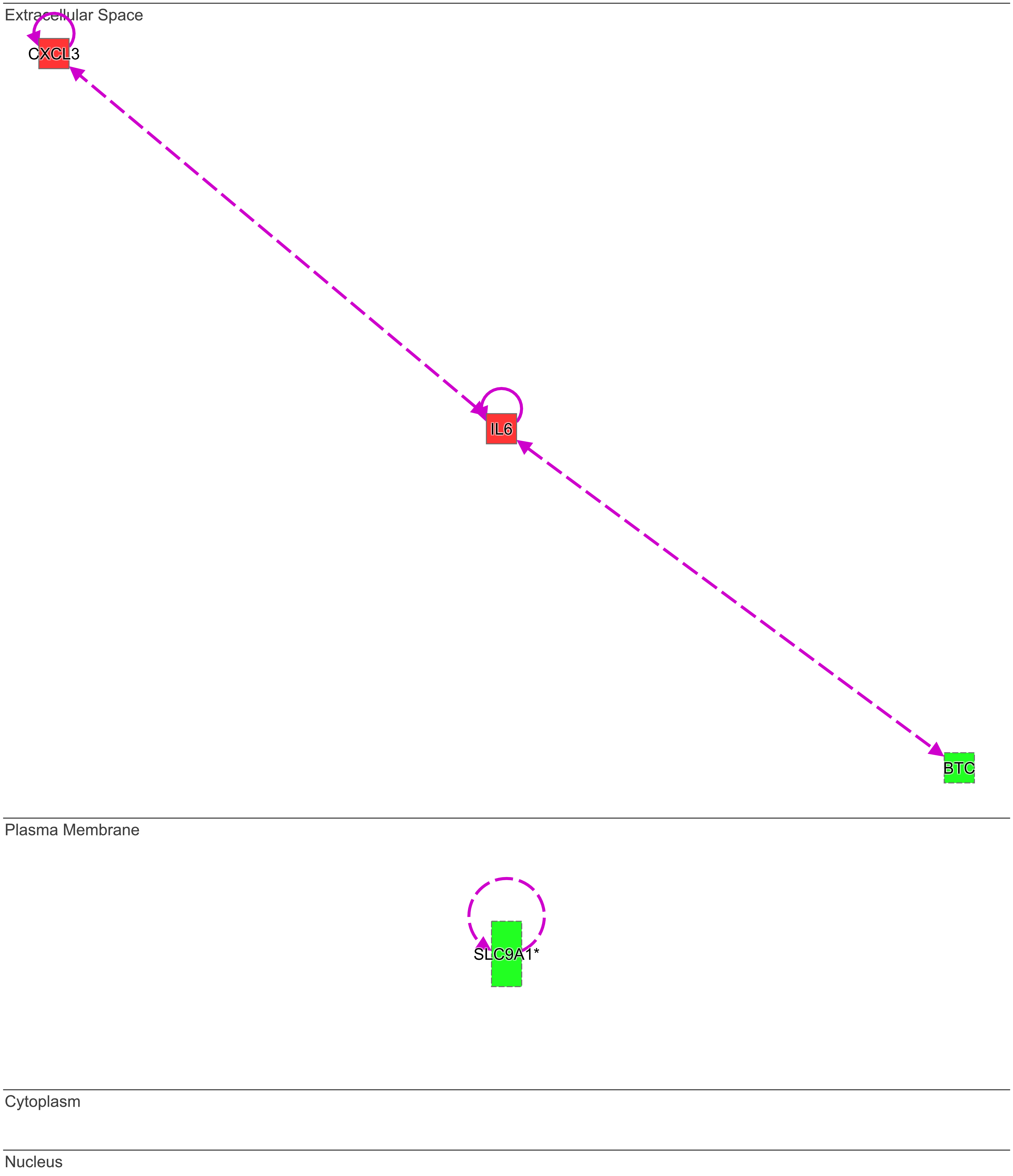
Extracellular Space
CXCL3
IL6
BTC
Plasma Membrane
SLC9A1*
Cytoplasm
Nucleus

1 hour

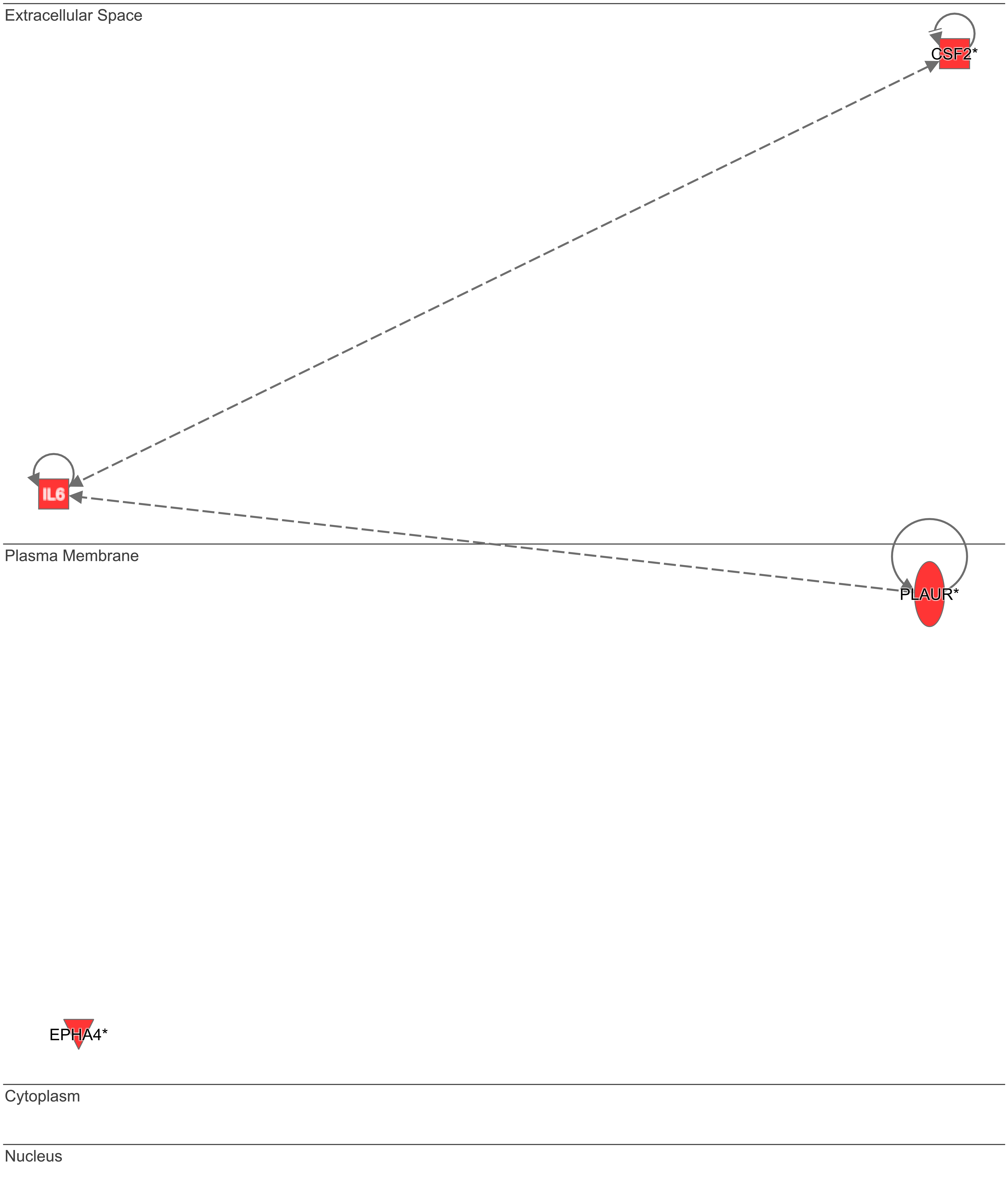

Extracellular Space
CSF2*
IL6
Plasma Membrane
PLAUR*
EPHA4*
Cytoplasm
Nucleus

6 hours

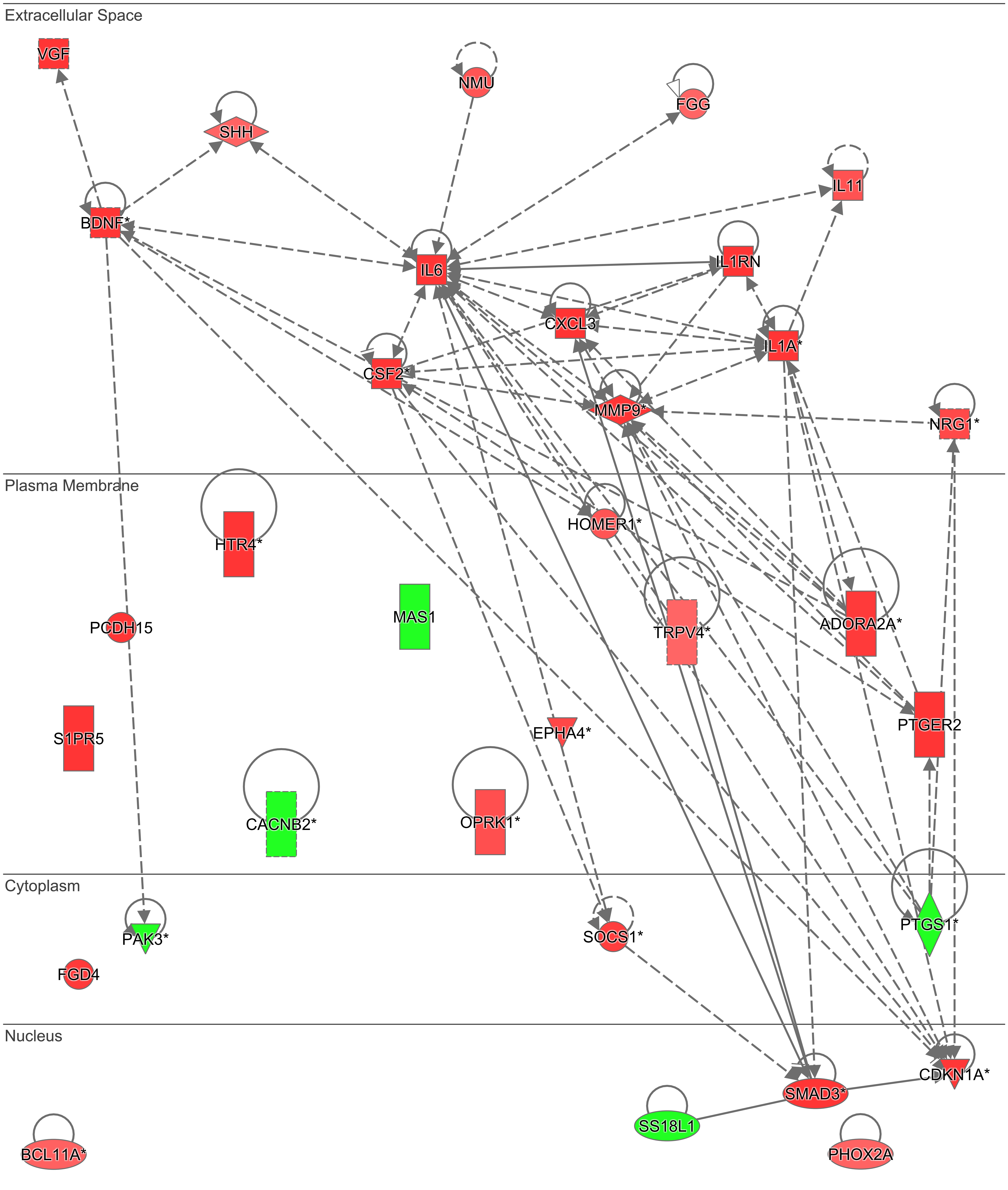
Extracellular Space
VGF
NMU
FGG
SHH
IL11
BDNF*
IL6
IL1RN
CXCL3
IL1A*
CSF2*
MMP9*
NRG1*
Plasma Membrane
HOMER1*
HTR4*
PCDH15
MAS1
TRPV4*
ADORA2A*
S1PR5
EPHA4*
PTGER2
CACNB2*
OPRK1*
Cytoplasm
PAK3*
SOCS1*
PTGS1*
FGD4
Nucleus
CDKN1A*
SMAD3*
SS18L1
BCL11A*
PHOX2A

12 hours

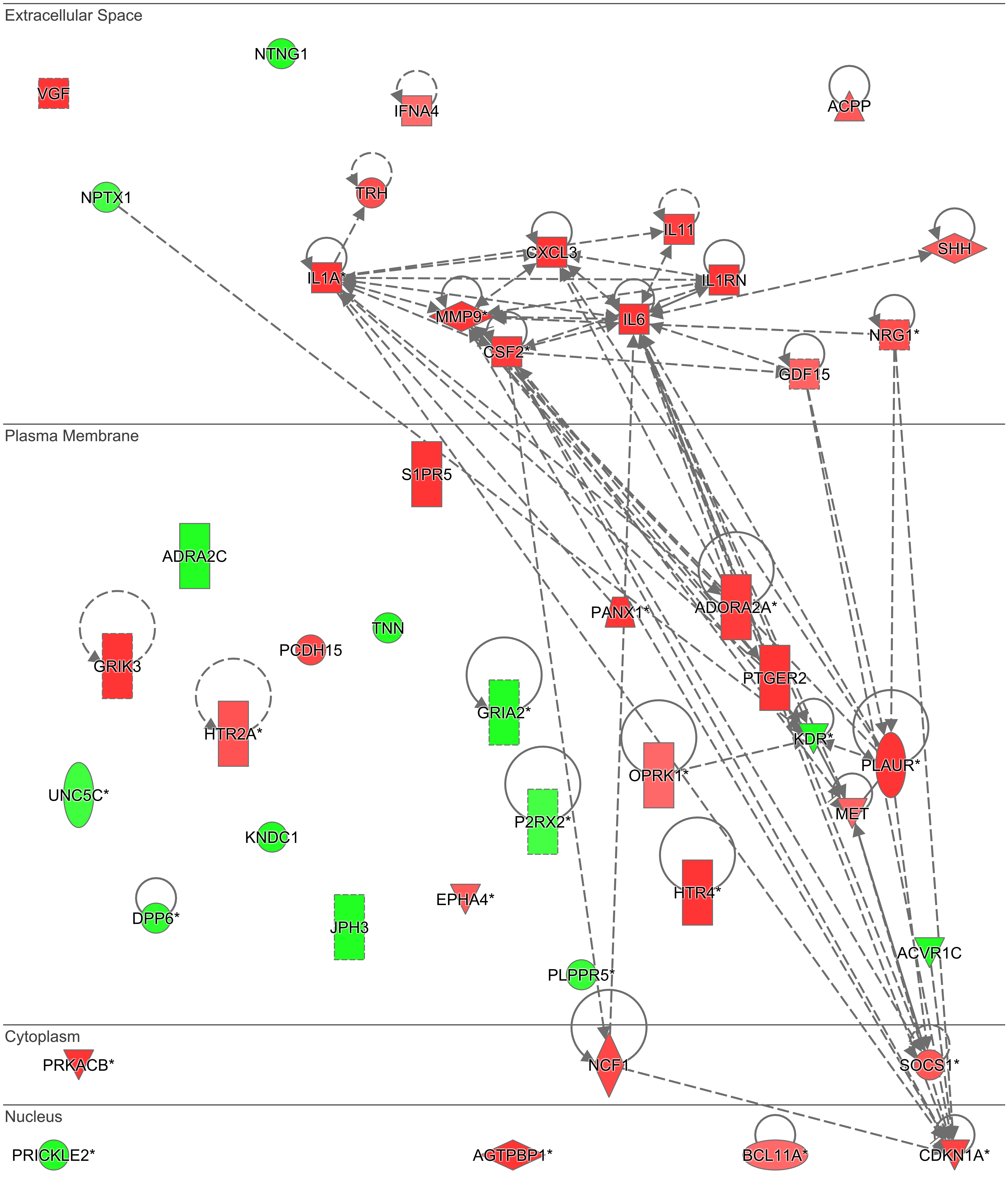
Extracellular Space
NTNG1
VGF
IFNA4
ACPP
NPTX1
TRH
IL11
CXCL3
SHH
IL1A*
IL1RN
MMP9*
IL6
NRG1*
CSF2*
GDF15
Plasma Membrane
S1PR5
ADRA2C
ADORA2A*
PANX1*
TNN
PCDH15
GRIK3
PTGER2
GRIA2*
HTR2A*
KDR*
PLAUR*
OPRK1*
UNC5C*
MET
P2RX2*
KNDC1
HTR4*
DPP6*
EPHA4*
JPH3
ACVR1C
PLPPR5*
Cytoplasm
PRKACB*
NCF1
SOCS1*
Nucleus
PRICKLE2*
AGTPBP1*
BCL11A*
CDKN1A*

24 hours

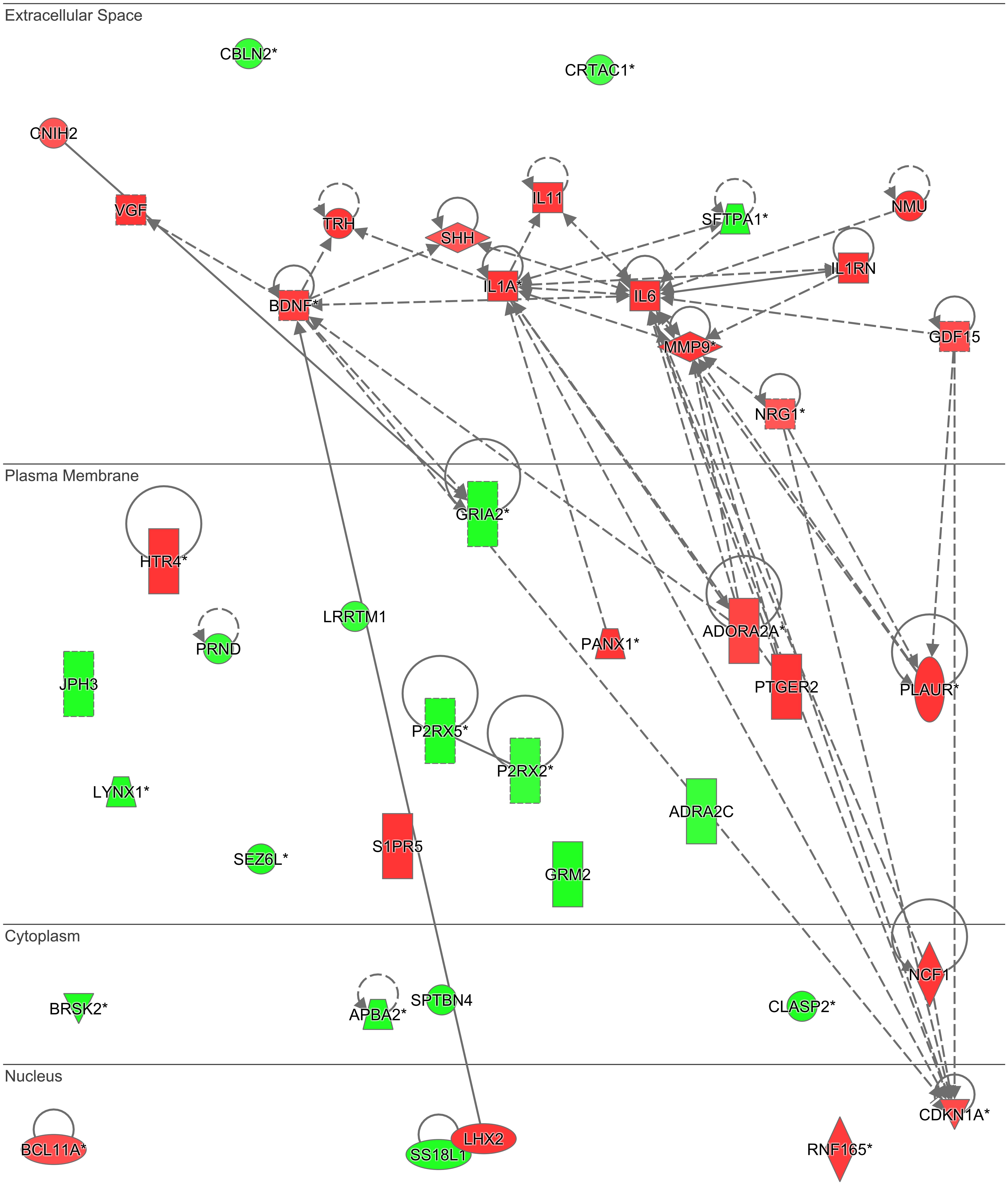
Extracellular Space
CBLN2*
CRTAC1*
CNIH2
VGF
TRH
SHH
IL11
SFTPA1*
NMU
IL1RN
IL1A*
IL6
BDNF*
MMP9*
GDF15
NRG1*
Plasma Membrane
GRIA2*
HTR4*
LRRTM1
PRND
PANX1*
ADORA2A*
PTGER2
PLAUR*
JPH3
P2RX5*
P2RX2*
LYNX1*
ADRA2C
S1PR5
SEZ6L*
GRM2
Cytoplasm
NCF1
BRSK2*
APBA2*
SPTBN4
CLASP2*
Nucleus
CDKN1A*
BCL11A*
LHX2
SS18L1
RNF165*

4 days

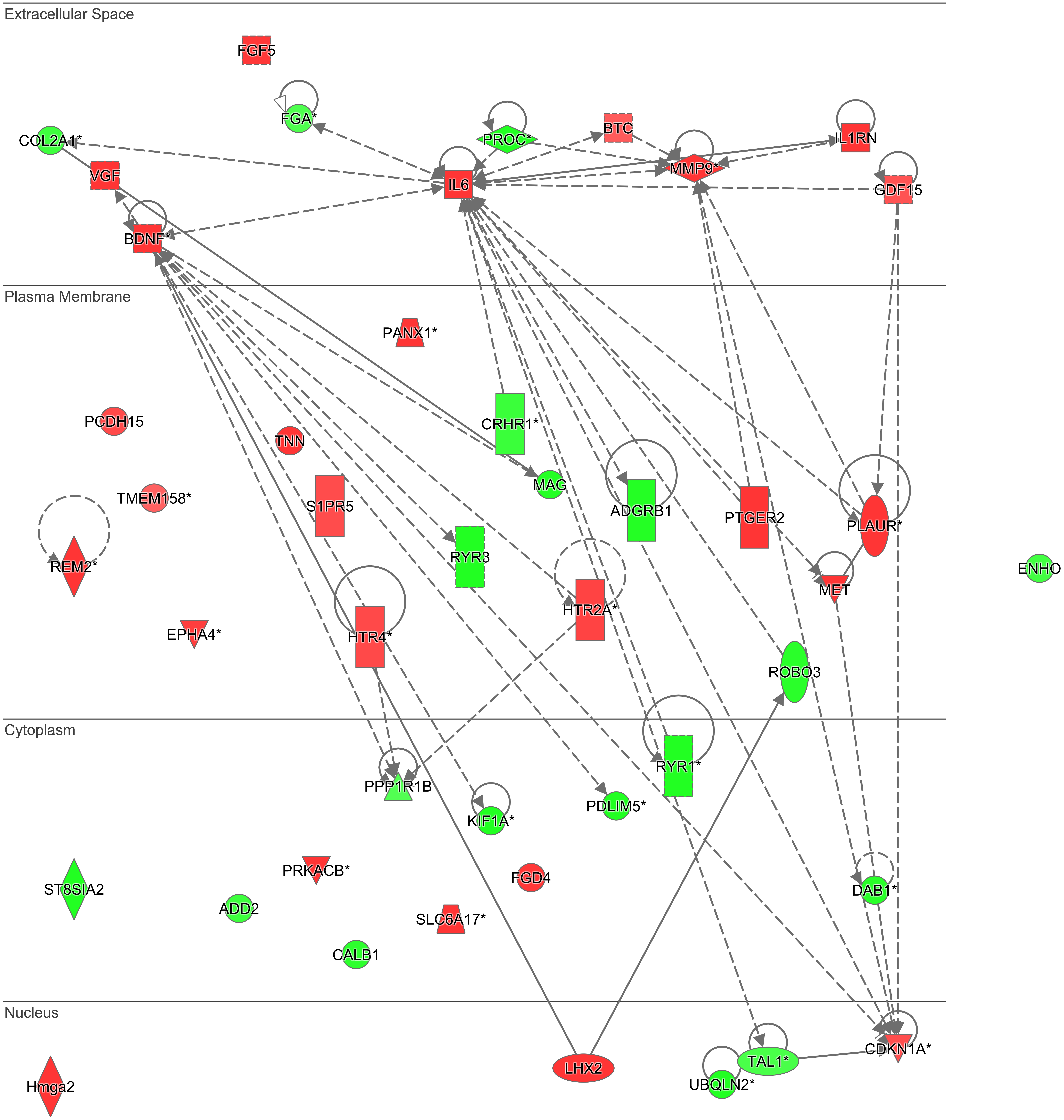

Extracellular Space
FGF5
FGA*
COL2A1*
PROC*
BTC
IL1RN
VGF
IL6
MMP9*
GDF15
BDNF*
Plasma Membrane
PANX1*
PCDH15
TNN
CRHR1*
MAG
TMEM158*
S1PR5
ADGRB1
PTGER2
PLAUR*
REM2*
RYR3
HTR2A*
MET
ENHO
EPHA4*
HTR4*
ROBO3
Cytoplasm
RYR1*
PPP1R1B
KIF1A*
PDLIM5*
PRKACB*
FGD4
DAB1*
ST8SIA2
ADD2
SLC6A17*
CALB1
Nucleus
LHX2
TAL1*
CDKN1A*
UBQLN2*
Hmga2

1 week

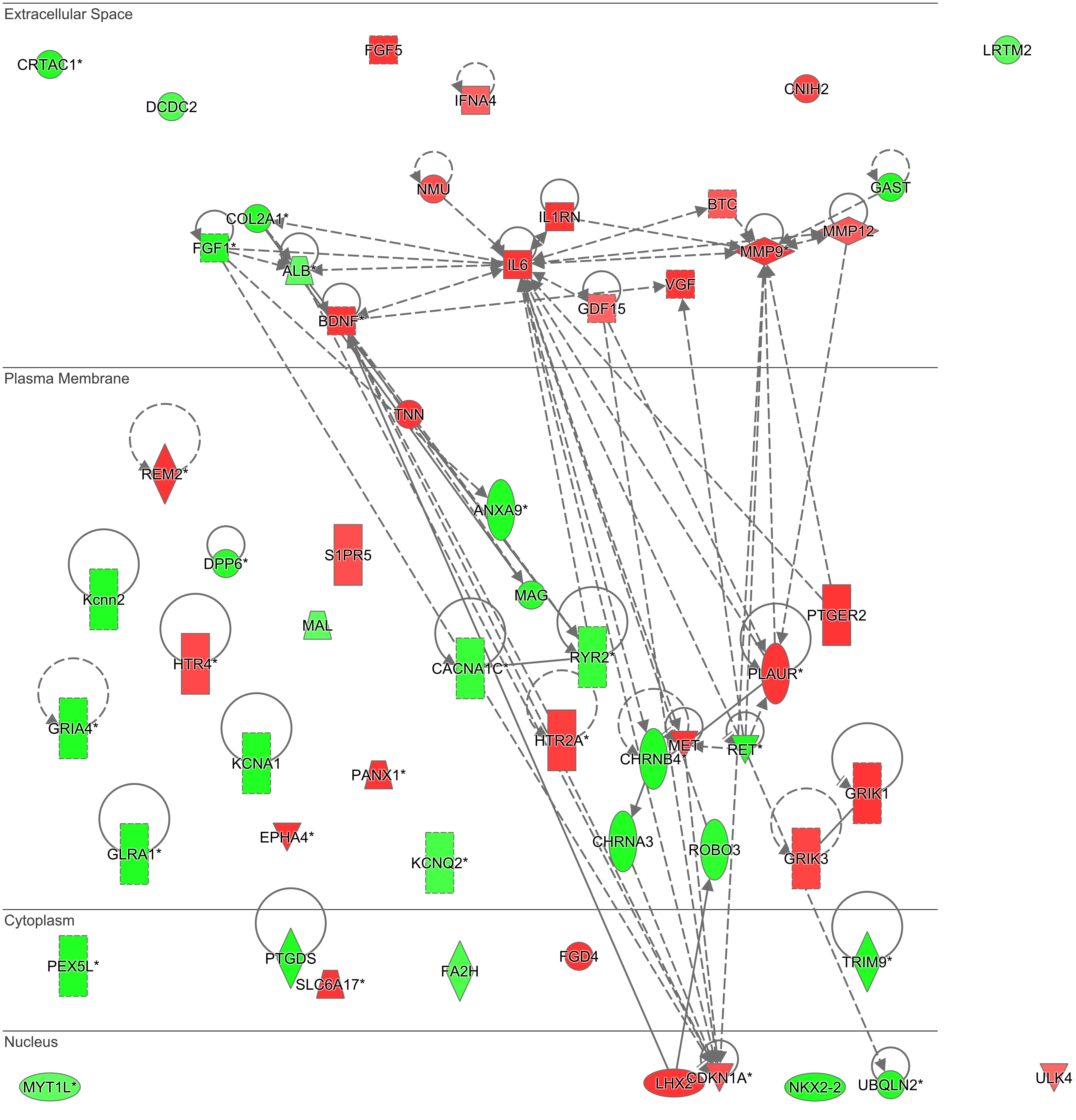
Extracellular Space
CRTAC1*
FGF5
LRTM2
DCDC2
IFNA4
CNIH2
NMU
IL1RN
BTC
GAST
COL2A1*
MMP12
FGF1*
IL6
MMP9*
ALB*
VGF
GDF15
BDNF*
Plasma Membrane
TNN
REM2*
ANXA9*
S1PR5
DPP6*
Kcnn2
MAG
PTGER2
MAL
HTR4*
RYR2*
CACNA1C*
PLAUR*
GRIA4*
HTR2A*
MET
RET*
CHRNB4*
KCNA1
PANX1*
GRIK1
GLRA1*
EPHA4*
CHRNA3
ROBO3
KCNQ2*
GRIK3
Cytoplasm
PEX5L*
PTGDS
FA2H
FGD4
TRIM9*
SLC6A17*
Nucleus
MYT1L*
LHX2
CDKN1A*
NKX2-2
UBQLN2*
ULK4

2 weeks

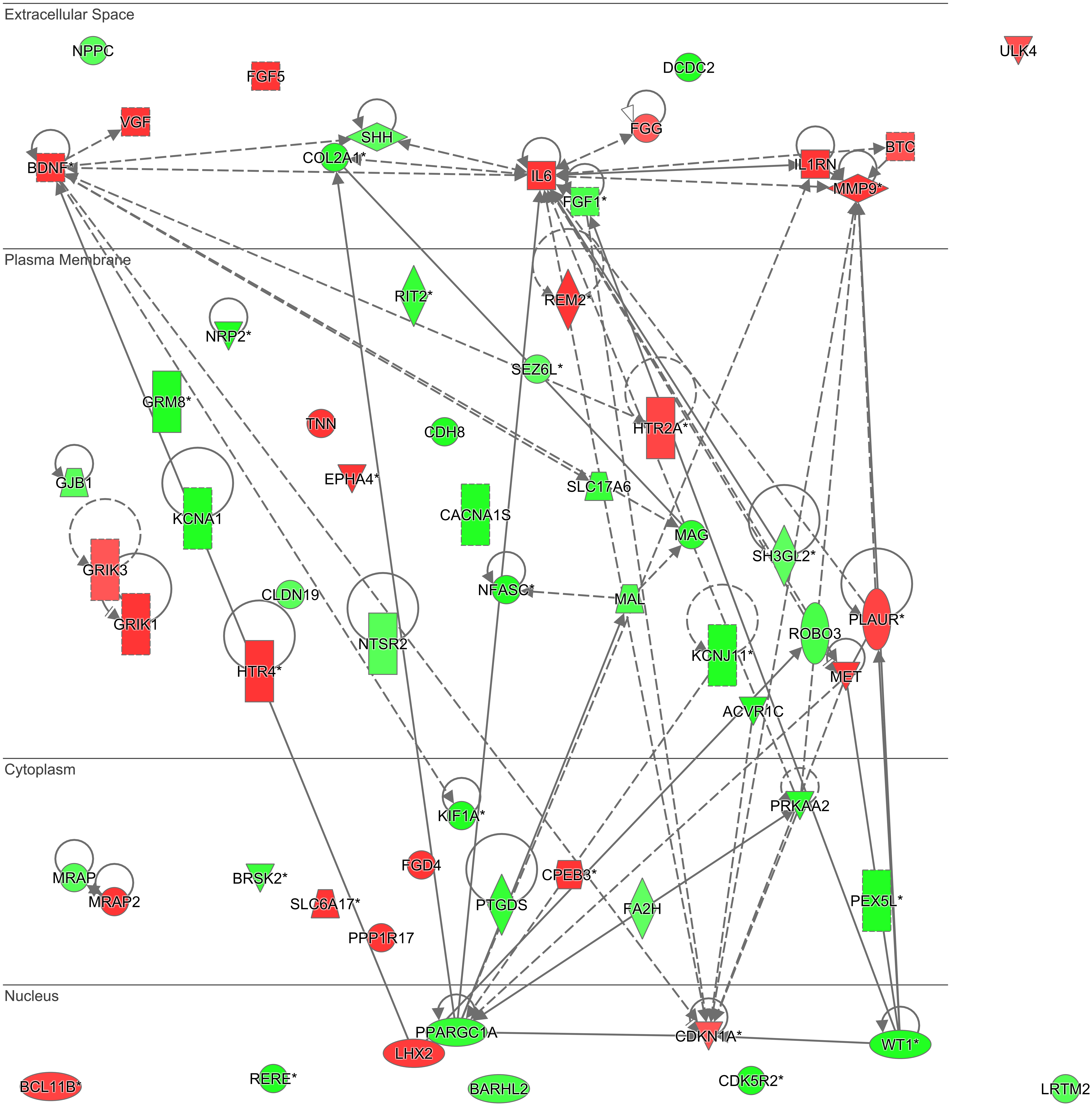

Extracellular Space
NPPC
FGF5
DCDC2
ULK4
VGF
SHH
FGG
BTC
BDNF*
COL2A1*
IL6
IL1RN
MMP9*
FGF1*
Plasma Membrane
RIT2*
REM2*
NRP2*
SEZ6L*
GRM8*
TNN
CDH8
HTR2A*
GJB1
EPHA4*
SLC17A6
CACNA1S
KCNA1
MAG
SH3GL2*
GRIK3
NFASC*
MAL
CLDN19
GRIK1
NTSR2
KCNJ11*
ROBO3
PLAUR*
HTR4*
MET
ACVR1C
Cytoplasm
PRKAA2
KIF1A*
MRAP
FGD4
CPEB3*
BRSK2*
MRAP2
SLC6A17*
PTGDS
FA2H
PEX5L*
PPP1R17
Nucleus
PPARGC1A
CDKN1A*
WT1*
LHX2
BCL11B*
RERE*
BARHL2
CDK5R2*
LRTM2

3 weeks

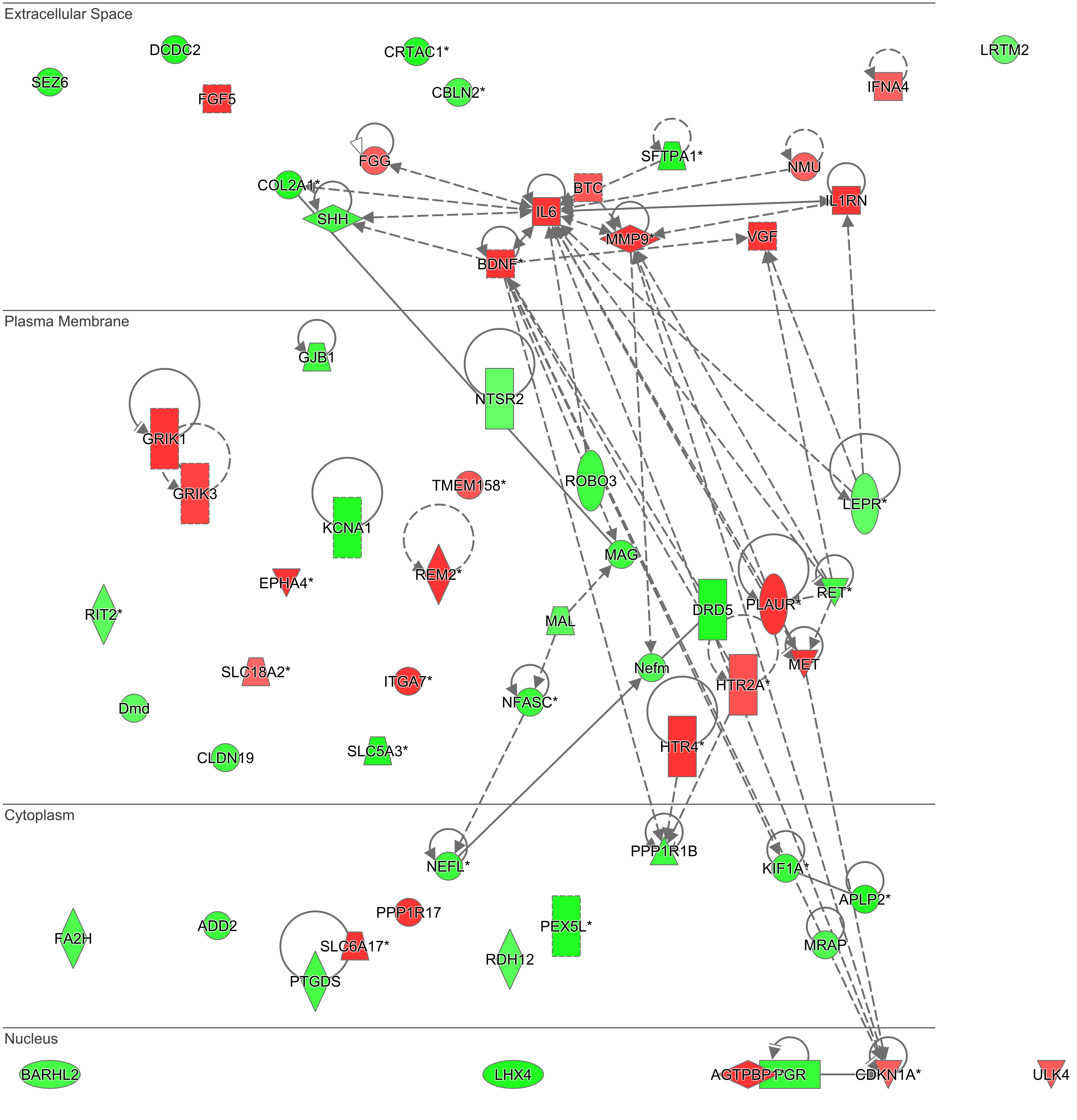
Extracellular Space
DCDC2
CRTAC1*
LRTM2
SEZ6
FGF5
CBLN2*
IFNA4
SFTPA1*
FGG
NMU
COL2A1*
BTC
IL1RN
IL6
SHH
MMP9*
VGF
BDNF*
Plasma Membrane
GJB1
NTSR2
GRIK1
GRIK3
TMEM158*
ROBO3
LEPR*
KCNA1
MAG
REM2*
EPHA4*
RET*
PLAUR*
DRD5
RIT2*
MAL
MET
SLC18A2*
ITGA7*
Nefm
NFASC*
HTR2A*
Dmd
HTR4*
CLDN19
SLC5A3*
Cytoplasm
PPP1R1B
NEFL*
KIF1A*
APLP2*
PPP1R17
PEX5L*
ADD2
FA2H
SLC6A17*
MRAP
RDH12
PTGDS
Nucleus
BARHL2
LHX4
AGTPBP1*
GR
CDKN1A*
ULK4
